# Supplementary material for: p53 enhances DNA repair and suppresses cytoplasmic chromatin fragments and inflammation in senescent cells
Source: Nat Commun. 2025 Mar 5;16:2229. doi: 10.1038/s41467-025-57229-3 (PMC11882782; doi:10.1038/s41467-025-57229-3)
Supplement: Supplementary file 1 — Supplementary Information [file 41467_2025_57229_MOESM1_ESM.pdf]

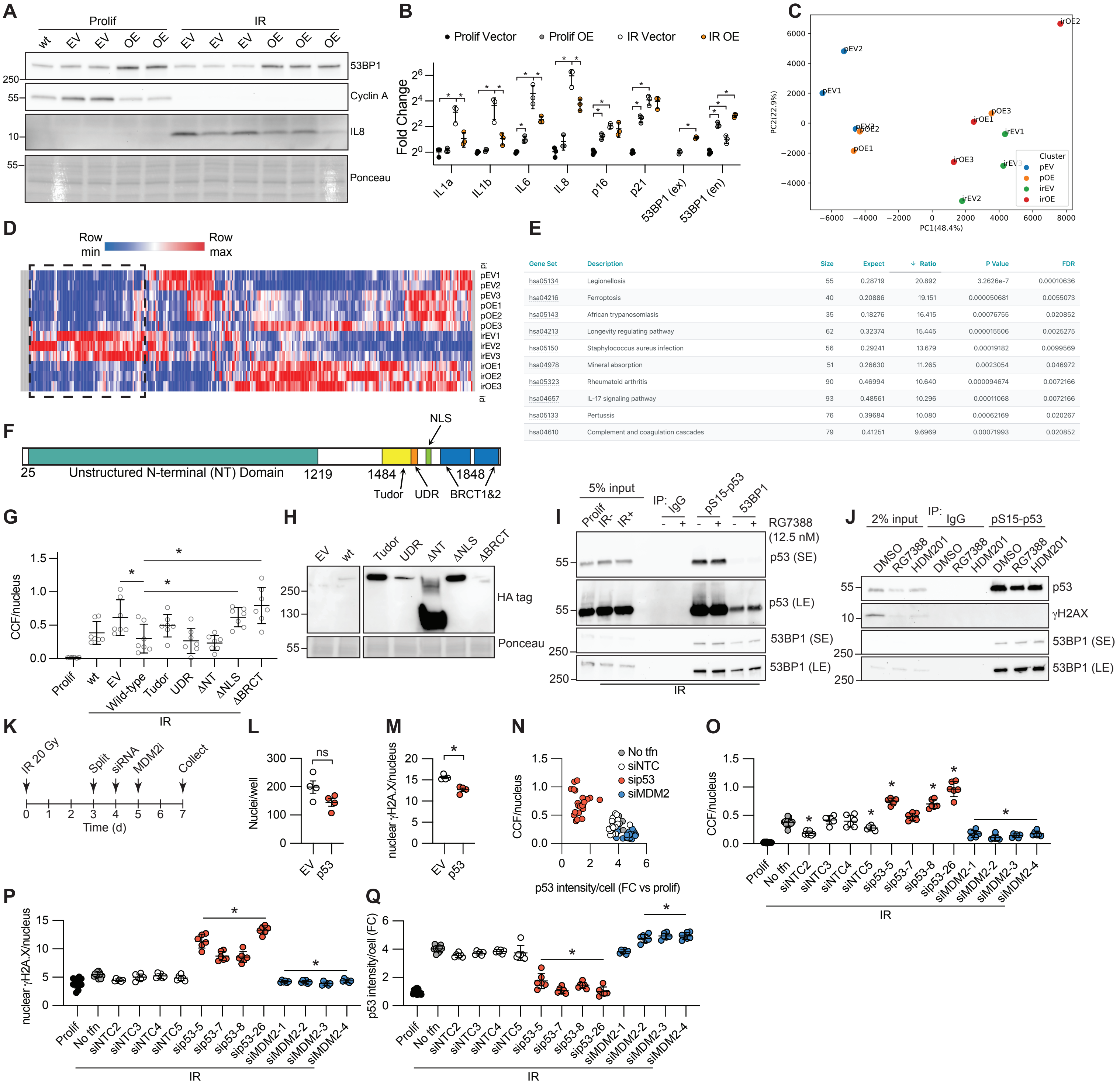

Figure S1: Related to Figure 1. A) WB representative of n=2 experiments and B) qPCR of 53BP1 and senescence markers in irradiation-induced senescent IMR90 human fibroblasts ectopically expressing 53BP1 or empty vector. C) RNA-seq principal component analysis, D) hierarchical clustering of differentially expressed genes, and E) KEGG ontology of cluster indicated by dashed box. F) 53BP1 protein map with major domains targeted by mutagenesis. G) CCF formation by IF representative of n=2 experiments of 53BP1 mutant protein expression in irradiation-induced senescent IMR90 human fibroblasts ectopically expressing wild-type or mutant 53BP1 or empty vector, and H) WB from a single experiment. I,J) IP of pS15-p53 or 53BP1 in irradiation-induced senescent IMR90 human fibroblasts, n=1 experiment each. γH2A.X blot is a wider crop of the same blot shown in Fig.2C. K) Schematic of siRNA experiments. L) Cell number and M) nuclear γH2A.X foci by IF related to Fig.1D, representative of n=3 experiments. N) Deconvolution of siRNA pools used in Fig.1B showing correlation between CCF/nucleus and p53 intensity per cell, and O-Q), assessing CCF, p53, and nuclear γH2A.X by IF, n=1 experiment. Data shown as means ±SD, asterisk(*) indicates p<0.05 by two-tailed Student's t-test. Statistical comparisons in M-P are to siNTC-3. Prolif: proliferating control; IR: ionizing radiation-induced senescence; wt: wild-type; EV: empty vector; OE: overexpression; en: endogenous transcript; ex: exogenous transcript; NTC: non-targeting control; NT: n-terminal domain; UDR: ubiquitin-dependent region; NLS: nuclear localization sequence; BRCT: Brca1 C-terminal sequence motif. SE/LE: short/long exposure. Source data are provided as a Source Data file.

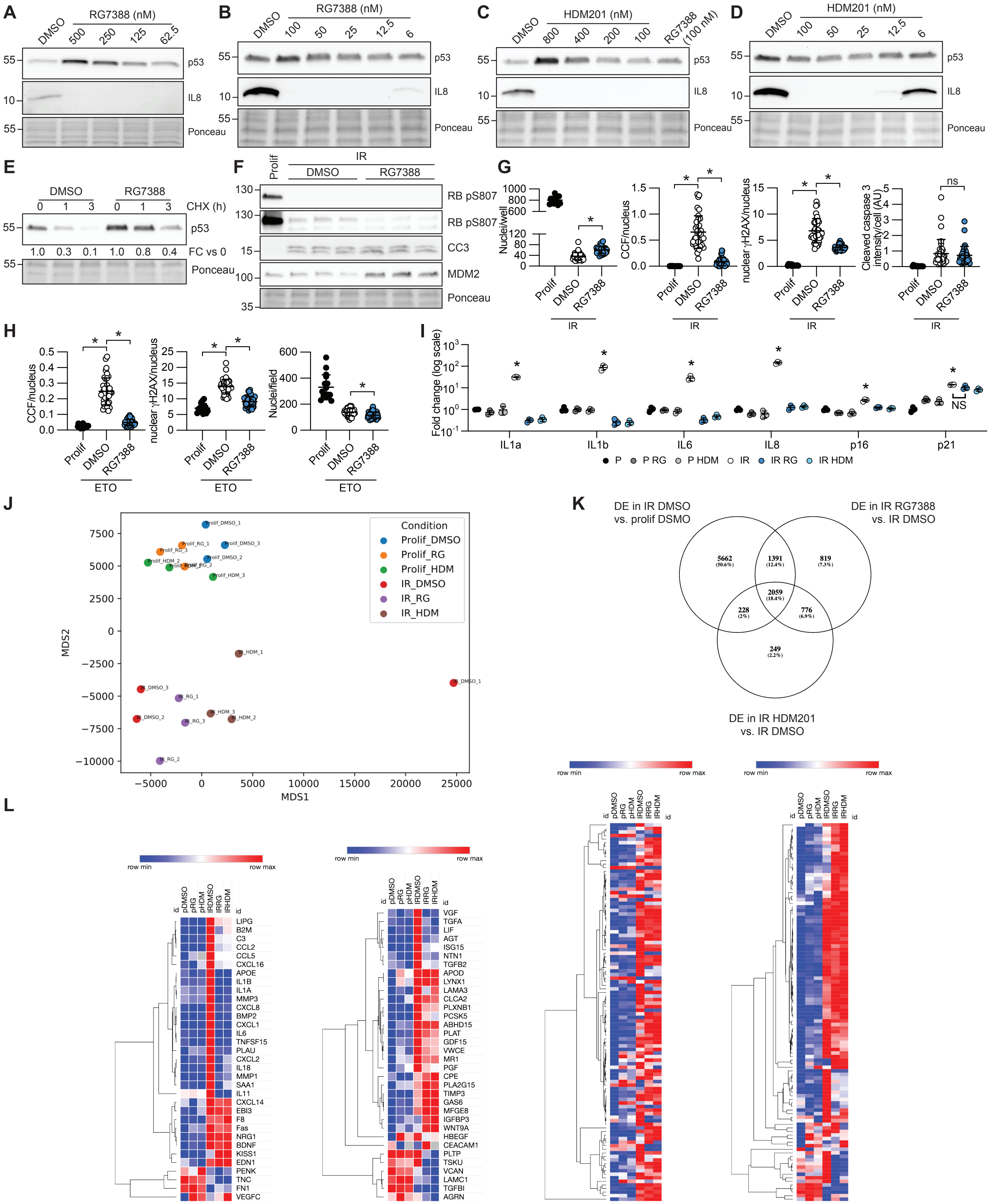
A
DMSO
RG7388 (nM)
500
250
125
62.5
p53
IL8
Ponceau
B
RG7388 (nM)
100
50
25
12.5
6
C
HDM201 (nM)
800
400
200
100
RG7388 (100 nM)
D
HDM201 (nM)
E
DMSO
RG7388
CHX (h)
FC vs 0
1.0 0.3 0.1 1.0 0.8 0.4
F
Prolif
IR
RB pS807
CC3
MDM2
G
Nuclei/well
CCF/nucleus
nuclear γH2AX/nucleus
Cleaved caspase 3 intensity/cell (AU)
ns
H
ETO
Nuclei/field
I
Fold change (log scale)
IL1a
IL1b
IL6
IL8
p16
p21
NS
P
P RG
P HDM
IR
IR RG
IR HDM
J
Condition
Prolif_DMSO
Prolif_RG
Prolif_HDM
IR_DMSO
IR_RG
IR_HDM
MDS1
MDS2
K
DE in IR DMSO vs. prolif DSMO
DE in IR RG7388 vs. IR DMSO
DE in IR HDM201 vs. IR DMSO
5662 (50.6%)
1391 (12.4%)
819 (7.3%)
2059 (18.4%)
228 (2%)
776 (6.9%)
249 (2.2%)
L
row min
row max
id
pDMSO
pRG
pHDM
IRDMSO
IRRG
IRHDM
LIPG
B2M
C3
CCL2
CCL5
CXCL16
APOE
IL1B
IL1A
MMP3
CXCL8
BMP2
CXCL1
IL6
TNFSF15
PLAU
CXCL2
IL18
MMP1
SAA1
IL11
CXCL14
EBI3
F8
Fas
NRG1
BDNF
KISS1
EDN1
PENK
TNC
FN1
VEGFC
VGF
TGFA
LIF
AGT
ISG15
NTN1
TGFB2
APOD
LYNX1
LAMA3
CLCA2
PLXNB1
PCSK5
ABHD15
PLAT
GDF15
VWCE
MR1
PGF
CPE
PLA2G15
TIMP3
GAS6
MFGE8
IGFBP3
WNT9A
HBEGF
CEACAM1
PLTP
TSKU
VCAN
LAMC1
TGFBI
AGRN

Figure S2: Related to Figure 1. A-D) MDM2i dose-response curves in irradiation-induced senescent IMR90 human fibroblasts by IL8 WB, n=1 experiment each. E) Cycloheximde chase assay in IMR90 cells 4 days after irradiation, measuring p53 protein level by WB and quantitation as fold change vs. 0 h for each group, representative of n=2 experiments. F) Irradiation-induced senescent IMR90 fibroblasts treated with 12.5 nM RG7388 for 14 days, showing markers of senescence and p53 activation by western blot, including two exposures of RB pS807, and G) cell number, CCF formation, nuclear γH2A.X, and cleaved caspase 3 intensity by IF, representative of n=2 experiments. H) Etoposide-induced senescent IMR90 fibroblasts showing CCF formation, nuclear γH2A.X, and cell number, representative of n=2 experiments. I) qPCR validation and J) RNA-seq principal component analysis for MDM2i treatment related to Fig.1G. K) Venn diagram of DE genes. L) Left to right: NFkB, p53, p21, and p16-associated secretomes. Data shown as means ±SD, asterisk(*) indicates $p<0.05$ by two-tailed Student's t-test. P or Prolif: proliferating control; IR: ionizing irradiation-induced senescence; CHX: cycloheximide; CC3: cleaved caspase 3; ETO: etoposide; RG: RG7388; HDM: HDM201. Source data are provided as a Source Data file.

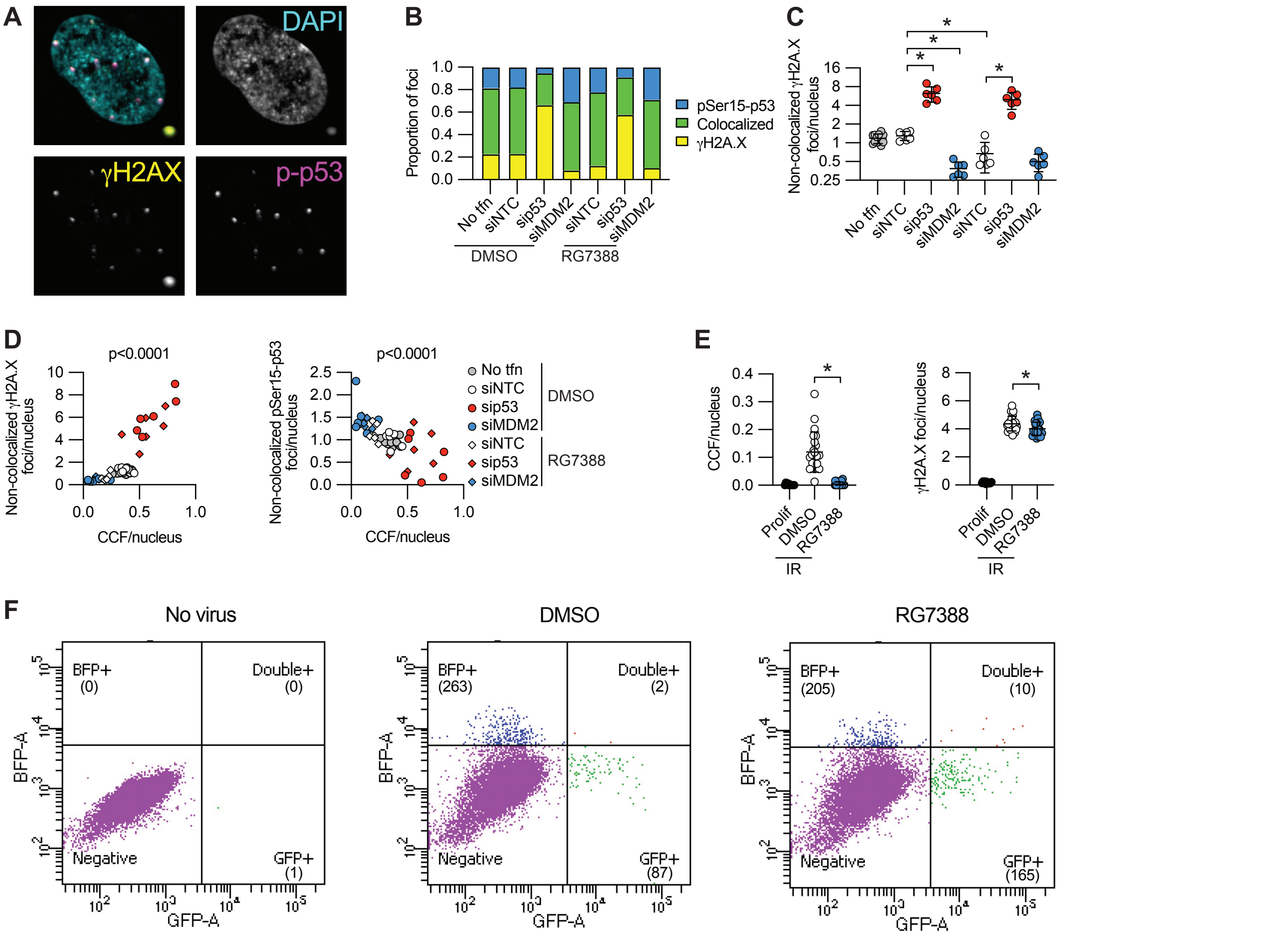

Figure S3: Related to Figure 2. A) Representative IF image of γH2A.X and pS15-p53 colocalization in irradiation-induced senescent IMR90 human fibroblasts and B) IF quantitation of colocalization between pS15-p53 and γH2A.X, representative of n=2 experiments. C) Quantitation of γH2A.X foci not colocalized with pS15-p53, and D) correlations to CCF formation of (left) γH2A.X foci not colocalized with pS15-p53 foci and (right) pS15-p53 foci not colocalized with γH2A.X foci, from same dataset as Fig.1B. E) Irradiation-induced senescent I9A human fibroblasts, showing CCF formation and nuclear γH2A.X foci by IF, representative of n=2 experiments. F) Representative raw data related to Fig.2G. Data shown as means ±SD, asterisk(*) indicates $p<0.05$ by two-tailed Student's t-test. Prolif: proliferating control; NTC: non-targeting control; BFP: blue fluorescent protein; GFP: green fluorescent protein. Source data are provided as a Source Data file.

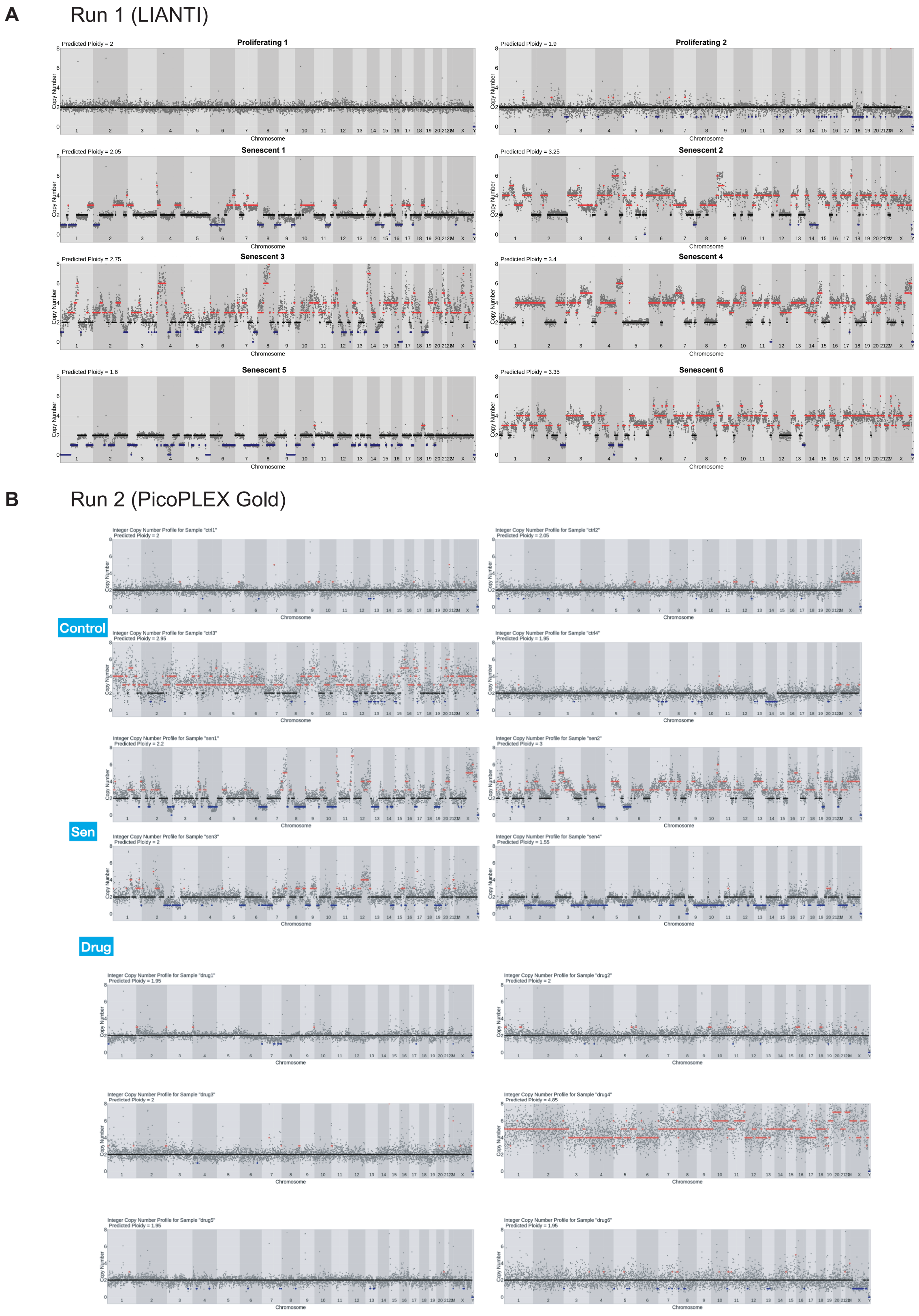

Figure S4: Related to Figure 3. Whole genome copy number variation plots for all cells sequenced by A) LIANTI or B) PicoPLEX Gold approaches. For B, Control refers to Proliferating control cells, Sen refers to ionizing radiation-induced senescent cells, and Drug refers to RG7388 treated ionizing radiation-induced senescent cells.

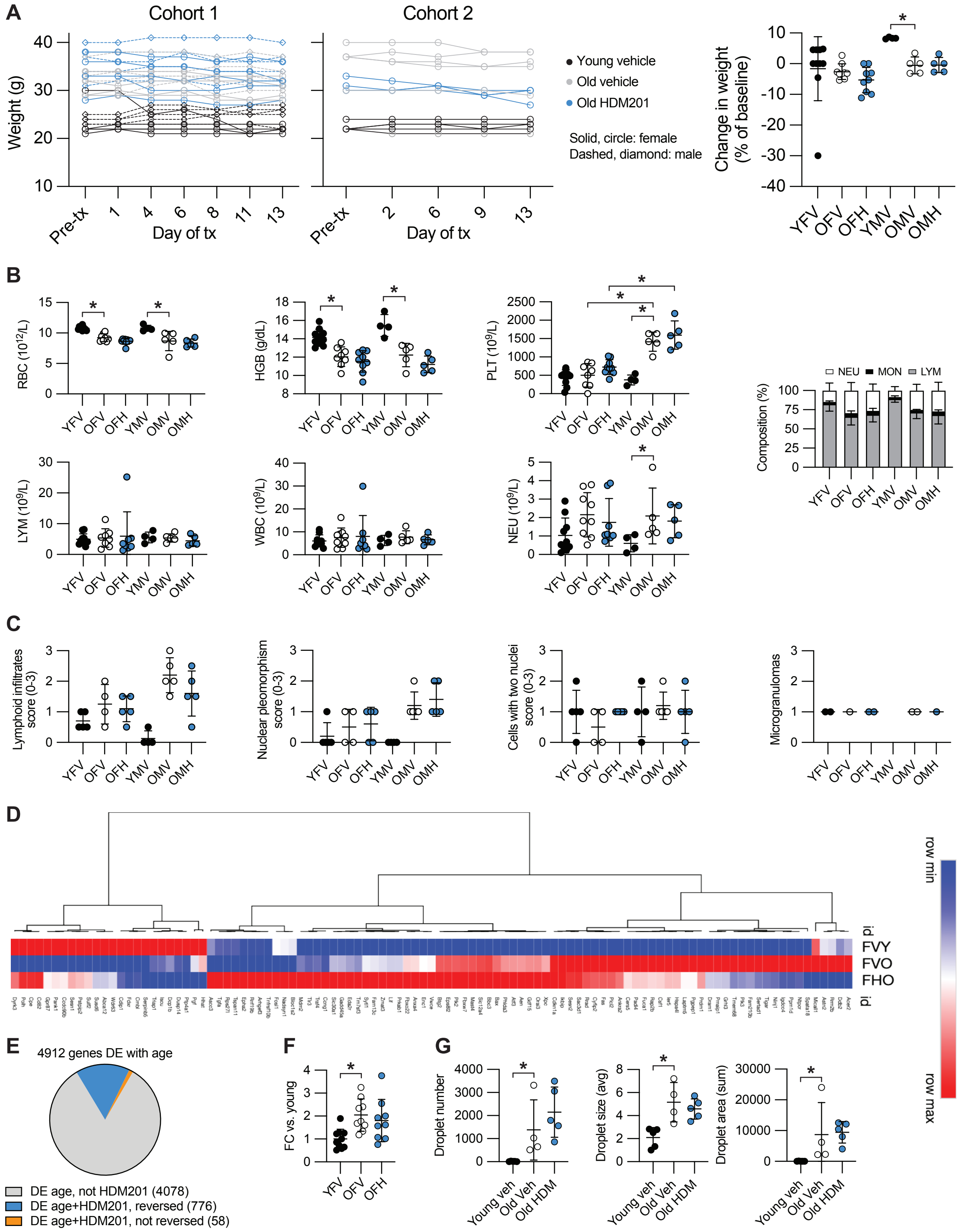

Figure S5: Related to Figure 4. A) Body weight and change in weight over the course of treatment as a percentage of baseline animal weight, n=10, 9, 9, 4, 5, 5 mice per group. B) Whole blood measures and white blood cell composition, n=10, 9, 9, 4, 5, 5 per group. C) Liver pathology indices scored by a trained pathologist, n=5, 4, 5, 4, 5, 5 mice per group. D) p53 target gene expression in female mouse liver by RNA-seq, n=5, 4, 5 mice per group. E) Comparison of genes differentially expressed with age and HDM201 in liver by bulk RNA-seq. F) Picrosirius red staining in female mice, n=10, 9, 9 mice per group. G) Oil red-O staining, n=5, 4, 5 mice per group. Data shown as means ±SD, asterisk(*) indicates $p<0.05$ by two-tailed Mann-Whitney U test. V: vehicle; H: HDM201; Y: young; O: old; F: female; M: male; tx: treatment; RBC: red blood cell; HGB: hemoglobin; PLT: platelet; LYM: lymphocyte; WBC: white blood cell; NEU: neutrophil; DE: differentially expressed. Source Data are provided as a Source Data file.

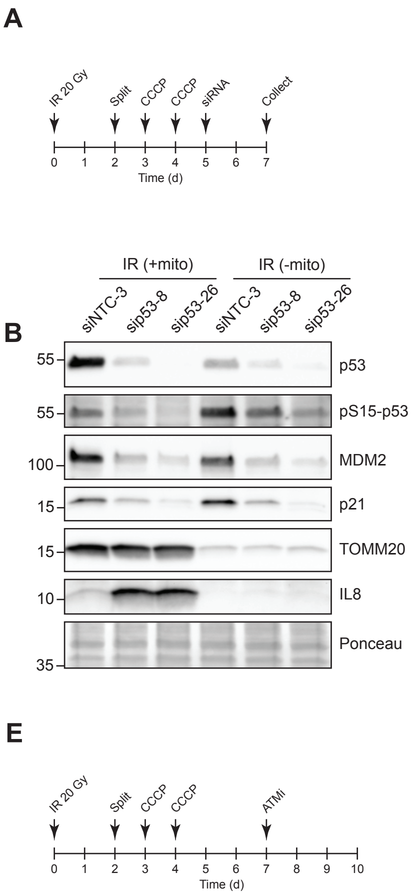

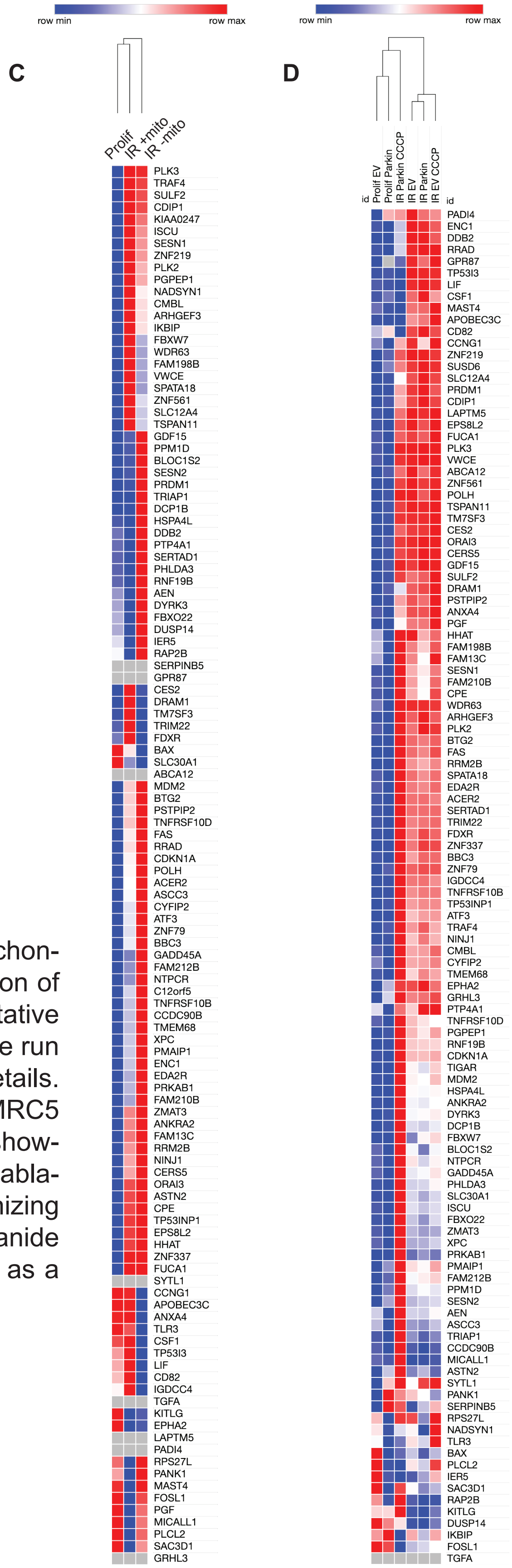

Figure S6: Related to Figure 5. A) Timeline for mitochondrial ablation experiments in Fig.5 A,B. B) WB validation of mitochondrial ablation and p53 knockdown representative of n=2 experiments, related to Fig.5A,B. The blots were run on two separate gels; see the Source Data for more details. C) p53 target genes with mitochondrial ablation in MRC5 fibroblasts, from ref51. D) Heatmap related to Fig.5C, showing additional controls. E) Timeline for mitochondrial ablation experiment with ATMi treatment in Fig.5D. IR: ionizing radiation-induced senescence; CCCP: carbonyl cyanide m-chlorophenyl hydrazone. Source data are provided as a Source Data file.

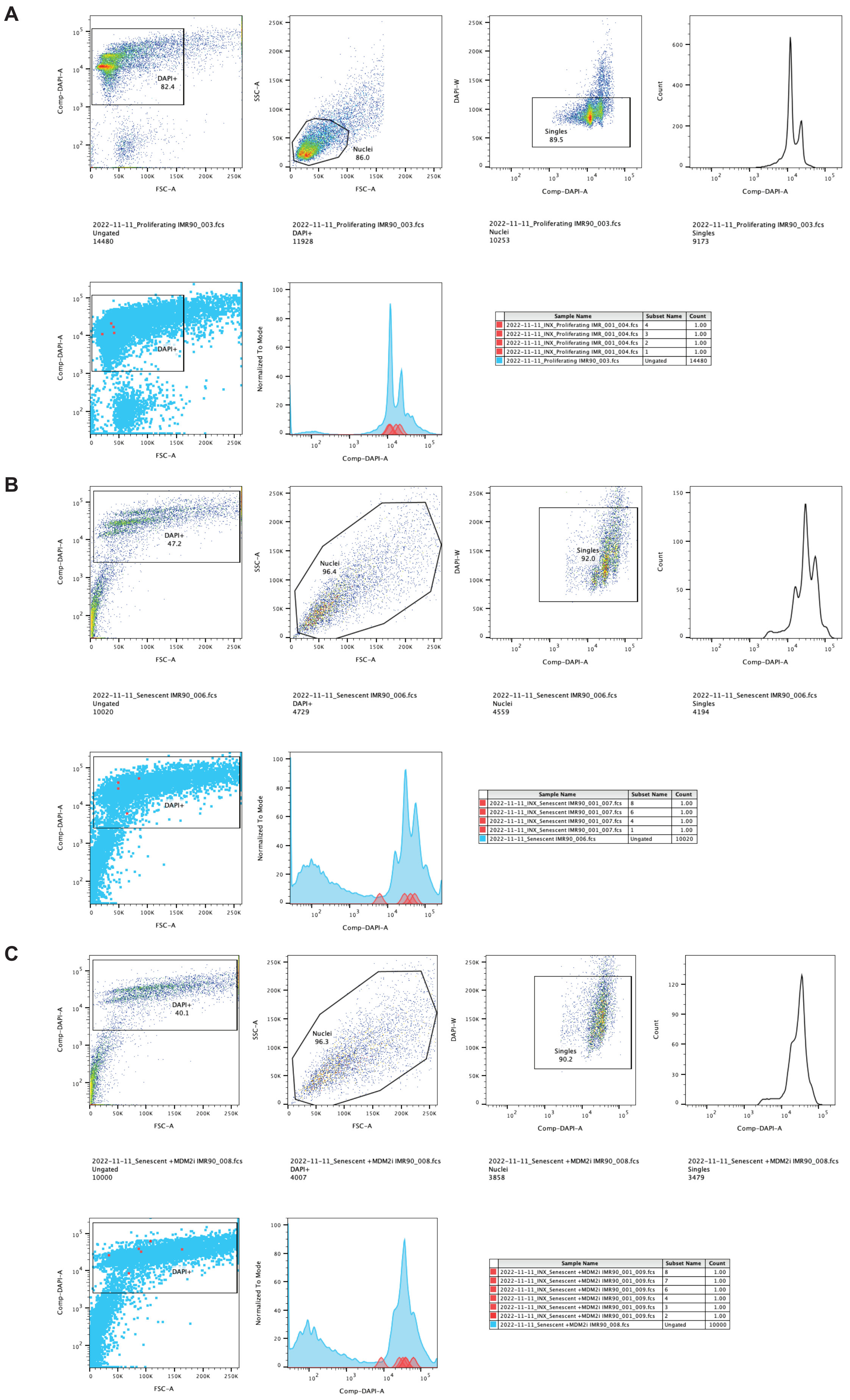

Figure S7: Gating of cell sorting for single-nucleus genome resequencing. A) Proliferating, B) Senescent, C) Senescent + MDM2i. Red marks correspond to the individual sorted nuclei from Fig.S4B.

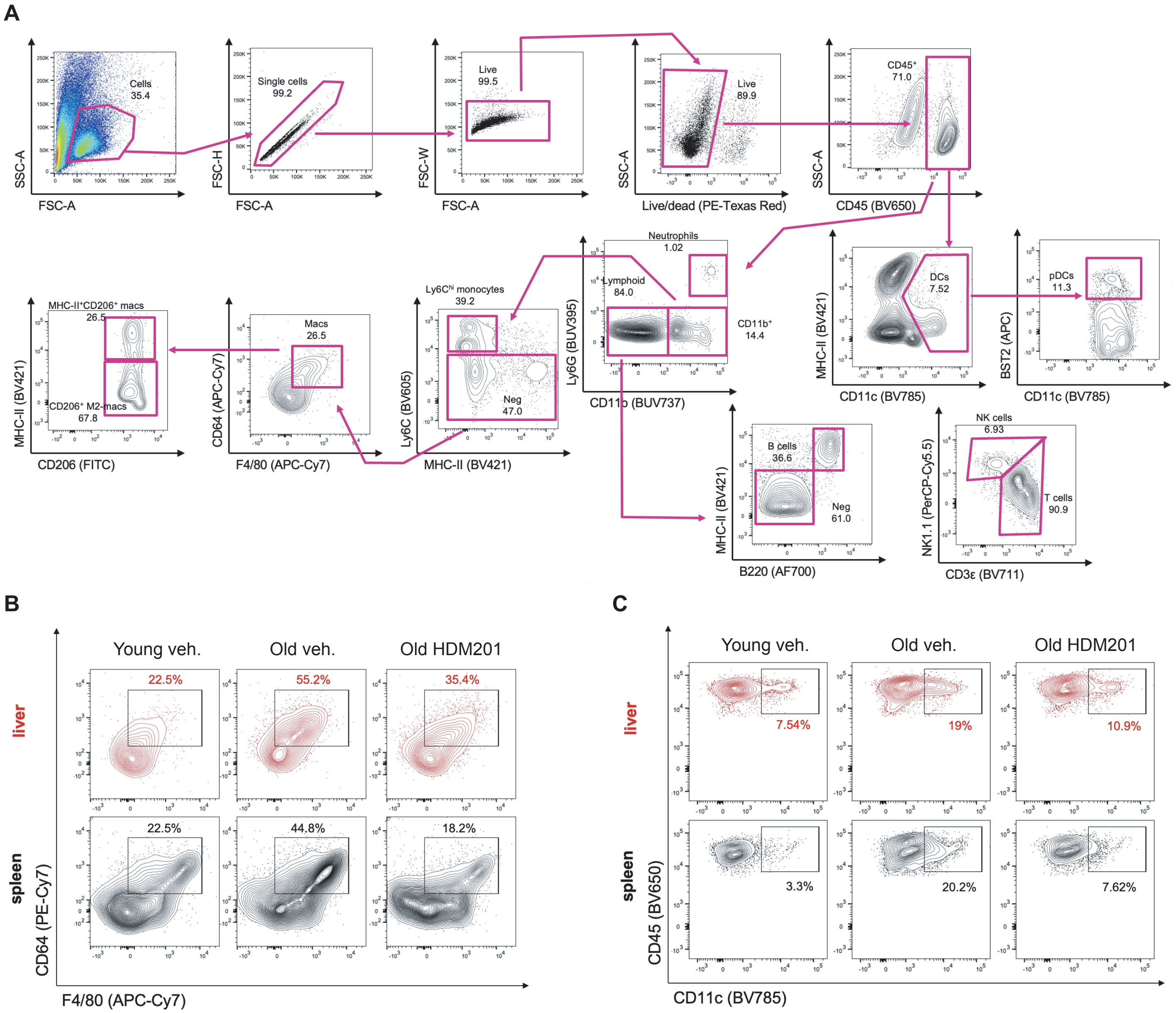

Figure S8: Flow cytometry gating for immune profiling related to Figure 4. A) Representative gating strategy for identifying immune cells mouse liver. B) Representative flow cytometry plots showing the frequency of macrophages (F4/80+CD64+) and C) dendritic cells (CD11c+) isolated from liver and spleen. The plots in B and C are gated on CD45+CD11b+Ly6C- cells.
