# Supplementary material for: p53 enhances DNA repair and suppresses cytoplasmic chromatin fragments and inflammation in senescent cells
Source: Nat Commun. 2025 Mar 5;16:2229. doi: 10.1038/s41467-025-57229-3 (PMC11882782; doi:10.1038/s41467-025-57229-3)
Supplement: Supplementary file 2 — Description of Additional Supplementary Information [file 41467_2025_57229_MOESM2_ESM.docx]

**Description of Additional Supplementary Files**

File Name: Supplementary Data 1

Description: RNAseq analysis from cell culture experiments.

File Name: Supplementary Data 2

Description: RNAseq analysis from mouse experiments.

File Name: Supplementary Data 3

Description: List of antibodies used for immune cell profiling.

File Name: Supplementary Data 4

Description: List of qPCR primers.
