# Supplementary material for: p53 enhances DNA repair and suppresses cytoplasmic chromatin fragments and inflammation in senescent cells
Source: Nat Commun. 2025 Mar 5;16:2229. doi: 10.1038/s41467-025-57229-3 (PMC11882782; doi:10.1038/s41467-025-57229-3)
Supplement: Supplementary file 7 — Reporting Summary [file 41467_2025_57229_MOESM7_ESM.pdf]

nature portfolio

Corresponding author(s): Karl Miller, Peter Adams

Last updated by author(s): Jan 31, 2025

Reporting Summary

Nature Portfolio wishes to improve the reproducibility of the work that we publish. This form provides structure for consistency and transparency in reporting. For further information on Nature Portfolio policies, see our Editorial Policies and the Editorial Policy Checklist.

Statistics

For all statistical analyses, confirm that the following items are present in the figure legend, table legend, main text, or Methods section.

n/a	Confirmed	
☐	☒	The exact sample size (n) for each experimental group/condition, given as a discrete number and unit of measurement
☐	☒	A statement on whether measurements were taken from distinct samples or whether the same sample was measured repeatedly
☐	☒	The statistical test(s) used AND whether they are one- or two-sided<br>*Only common tests should be described solely by name; describe more complex techniques in the Methods section.*
☒	☐	A description of all covariates tested
☐	☒	A description of any assumptions or corrections, such as tests of normality and adjustment for multiple comparisons
☐	☒	A full description of the statistical parameters including central tendency (e.g. means) or other basic estimates (e.g. regression coefficient) AND variation (e.g. standard deviation) or associated estimates of uncertainty (e.g. confidence intervals)
☐	☒	For null hypothesis testing, the test statistic (e.g. F, t, r) with confidence intervals, effect sizes, degrees of freedom and P value noted<br>*Give P values as exact values whenever suitable.*
☒	☐	For Bayesian analysis, information on the choice of priors and Markov chain Monte Carlo settings
☒	☐	For hierarchical and complex designs, identification of the appropriate level for tests and full reporting of outcomes
☒	☐	Estimates of effect sizes (e.g. Cohen's d, Pearson's r), indicating how they were calculated

Our web collection on statistics for biologists contains articles on many of the points above.

Software and code

Policy information about availability of computer code

Data collection	Except for Figs.2F and 3D, immunofluorescence imaging was done using automated image capture in NIS Elements AR v5.21.03 in a predetermined pattern for each sample

Data analysis	For IF, images were analyzed in NIS Elements AR v5.21.03<br><br>For RNAseq analysis, raw fastq files were aligned to hg19 (53BP1 OE RNAseq) or hg38 (MDM2i in cell culture RNAseq), or mm10 (MDM2i in mice RNAseq) using STAR69 2-pass pipeline. Reads were filtered, sorted and indexed by SAMtools70. FPKM were generated using CuffLinks71 for downstream visualization. Genome tracks (bigWig files) were obtained by Deeptools72. Raw read counts were obtained by HTSeq73 for differential analysis. Differentially expressed genes were obtained by DESeq274. KEGG gene ontology was run using WebGestalt75, gene lists were compared using Venny2.1(https://bioinfogp.cnb.csic.es/tools/venny/index.html), and heatmaps were generated using Morpheus (https://software.broadinstitute.org/Morpheus).<br><br>For snDNA-seq analysis, the first 14 bases of both R1 and R2 reads were trimmed using CutAdapt76. Trimmed reads were then aligned to hg38 using Bowtie277. The sam files were transferred to bam files, then sorted and indexed using SAMtools70. Duplicates were removed using picard tools (https://broadinstitute.github.io/picard/) MarkDuplicates function. Copy number variation profiles were obtained via Ginkgo78<br><br>For flow cytometry, data were analyzed using FlowJo (v.10, BD Biosciences).<br><br>Automated comet analysis was performed using an open-source tool in ImageJ (Gyori et al 2014).

For manuscripts utilizing custom algorithms or software that are central to the research but not yet described in published literature, software must be made available to editors and reviewers. We strongly encourage code deposition in a community repository (e.g. GitHub). See the Nature Portfolio guidelines for submitting code & software for further information.

Data

Policy information about availability of data

All manuscripts must include a data availability statement. This statement should provide the following information, where applicable:

- Accession codes, unique identifiers, or web links for publicly available datasets
- A description of any restrictions on data availability
- For clinical datasets or third party data, please ensure that the statement adheres to our policy

Source data are provided with this paper. All sequencing data are deposited in GEO under accession code GSE259308. All other raw data can be found in the source data or can be obtained by contacting the corresponding authors.

Research involving human participants, their data, or biological material

Policy information about studies with human participants or human data. See also policy information about sex, gender (identity/presentation), and sexual orientation and race, ethnicity and racism.

Reporting on sex and gender	n/a
Reporting on race, ethnicity, or other socially relevant groupings	n/a
Population characteristics	n/a
Recruitment	n/a
Ethics oversight	n/a

Note that full information on the approval of the study protocol must also be provided in the manuscript.

Field-specific reporting

Please select the one below that is the best fit for your research. If you are not sure, read the appropriate sections before making your selection.

[x] Life sciences [ ] Behavioural & social sciences [ ] Ecological, evolutionary & environmental sciences

For a reference copy of the document with all sections, see nature.com/documents/nr-reporting-summary-flat.pdf

Life sciences study design

All studies must disclose on these points even when the disclosure is negative.

Sample size	Sample sizes were determined empirically on a per-experiment basis.
Data exclusions	For data generated by automated imaging, images that were out of focus or contained obvious technical artifacts were excluded.<br>For processed data, obvious outliers were verified by Grubb's test and removed. For cohort 1 of the animal experiments, one young male mouse was excluded from data analysis due to the presence of an open wound and suspected infection at time of collection.
Replication	For cell culture experiments, each biological replicate is one well of a culture plate (the smallest object that can be randomly and

independently assigned to an intervention), for example drug treatment or siRNA. For experiments in mice, each mouse was considered a biological replicate. All experiments were reproducible in 3/3, or in a few cases where noted, 2/2 experiments.

Randomization	Animals were randomly assigned into treatment groups. For all other experiments, randomization was not used.
Blinding	Blinding was used for initial sample processing and analysis for animal experiments, except for Fig.4A.<br>Blinding was not usually possible in cell culture experiments due to obvious differences in cell morphology and experimental outcome between groups. To limit bias, immunofluorescence imaging was done using automated image capture in NIS Elements AR v5.21.03 in a predetermined pattern for each sample, and all images were scored by NIS Elements AR v5.21.03 software to avoid bias of manual scoring, with the exceptions of Figs.2F and 3D.

Reporting for specific materials, systems and methods

We require information from authors about some types of materials, experimental systems and methods used in many studies. Here, indicate whether each material, system or method listed is relevant to your study. If you are not sure if a list item applies to your research, read the appropriate section before selecting a response.

Materials & experimental systems

n/a	Involved in the study
☐	☒ Antibodies
☐	☒ Eukaryotic cell lines
☐	☐ Palaeontology and archaeology
☐	☒ Animals and other organisms
☐	☐ Clinical data
☐	☐ Dual use research of concern
☐	☐ Plants

Methods

n/a	Involved in the study
☐	☐ ChIP-seq
☐	☒ Flow cytometry
☐	☐ MRI-based neuroimaging

Antibodies

Antibodies used	The following primary antibodies were used: 53BP1 (Cell Signaling Technology Cat#4937, RRID:AB_10694558), ATM (D2E2) (Cell Signaling Technology Cat#2873, RRID:AB_2062659), CENPA (Thermo Fisher Scientific Cat#MA1-20832, RRID:AB_2078763), Cyclin A (Santa Cruz Biotechnology Cat#sc-271682, RRID:AB_10709300), ANTI-FLAG M2 (Sigma-Aldrich Cat#F3165-2MG, RRID:AB_259529), Phospho-Histone H2A.X (Ser139) (Millipore Cat#05-636, RRID:AB_309864, Active Motif Cat#39117, RRID:AB_2793161), HA-probe (F-7) (Santa Cruz Biotechnology Cat#sc-7392, RRID:AB_627809), IgG control (Vector Laboratories Cat#I-1000, RRID:AB_2336355, Vector Laboratories Cat#I-2000, RRID:AB_2336354), IL8 (Abcam Cat#ab18672, RRID:AB_444617), MDM2 (Cell Signaling Technology Cat#51541, RRID:AB_2936381), p21 (Santa Cruz Biotechnology Cat#sc-817, RRID:AB_628072), p53 (Santa Cruz Biotechnology Cat#sc-126, RRID:AB_628082, Leica Biosystems Cat#NCL-p53-CM5p, RRID:AB_563933), Phospho-p53 (Ser15) (Cell Signaling Technology Cat#9284S, RRID:AB_331464), Phospho-ATM (Ser1981) (Abcam Cat#ab81292, RRID:AB_1640207), TOMM20 (Abcam Cat#ab56783, RRID:AB_945896). The following secondary antibodies were used: Goat anti-Mouse IgG, IgM (H+L) HRP (Thermo Fisher Scientific Cat#31446, RRID:AB_228318), Goat anti-Rabbit IgG, (H+L) HRP (Millipore Cat#AP307P, RRID:AB_92641), Goat anti-Mouse IgG (H+L), Alexa FluorTM 594 (Thermo Fisher Scientific Cat#A11032, RRID:AB_2534091), Goat anti-Rabbit IgG (H+L), Alexa FluorTM 488 (Thermo Fisher Scientific Cat#A11008, RRID:AB_143165).
Validation	The following antibodies were considered to be specific in our experimental system by RNAi against the target protein: 53BP1 (unpublished), MDM2 (Fig.1B), p21 (Fig.2E), p53 (Fig.1B), phospho-p53 (Ser15) (Fig.6B). The following antibodies were considered to be specific by (other methods): ANTI-FLAG M2 (Fig.1D) and HA-probe (no signal in empty vector) (Fig.S1G), phospho-histone H2A.X (staining pattern confirmed by a second antibody) (unpublished). The following antibodies were not rigorously confirmed, but have strong evidence supporting their specificity: Cyclin A (expression lost in senescent cells confirmed to exit the cell cycle by other markers) (Fig.S1A), IL8 (band at predicted MW is NFkB-dependent in senescent cells) (unpublished), TOMM20 (expression is lost with mitochondrial ablation that was confirmed by other markers) (Fig.S6B, Fig.5D, and unpublished). For immunofluorescence, specificity of the staining pattern was confirmed by comparing to an appropriate isotype control, and when possible also compared with a second antibody to the same target protein. All other targets were considered potentially specific based on other evidence, for example images in relevant published literature.

Eukaryotic cell lines

Policy information about cell lines and Sex and Gender in Research

Cell line source(s)	IMR90 (primary human, female) and 293T (immortalized/transformed) were purchased from the American Type Culture Collection (CCL-186 and CRL-3216 respectively). I9A (immortalized) were a gift from Dr. Vera Gobunova.
Authentication	Cell lines were not authenticated.
Mycoplasma contamination	Cells were regularly tested for mycoplasma contamination and scored negative.
Commonly misidentified lines<br>(See ICLAC register)	n/a

Palaeontology and Archaeology

Specimen provenance	n/a
Specimen deposition	n/a
Dating methods	n/a

☐ Tick this box to confirm that the raw and calibrated dates are available in the paper or in Supplementary Information.

Ethics oversight	n/a

Note that full information on the approval of the study protocol must also be provided in the manuscript.

Animals and other research organisms

Policy information about studies involving animals; ARRIVE guidelines recommended for reporting animal research, and Sex and Gender in Research

Laboratory animals	Cohort1: C57BL6J (old) and C57BL6N (young). Cohort 2: C57BL6J.
Wild animals	n/a
Reporting on sex	Experiments were carried out in both sexes. The effects of MDM2 inhibitor treatment with the regimen described were greater in female mice. Data on both sexes is shown in fig.S5 and table S2. See manuscript for more details.
Field-collected samples	n/a
Ethics oversight	This study was approved by the Institutional Animal Care and Use Committee at Sanford Burnham Prebys MDI.

Note that full information on the approval of the study protocol must also be provided in the manuscript.

Clinical data

Policy information about clinical studies

All manuscripts should comply with the ICMJEguidelines for publication of clinical research and a completedCONSORT checklist must be included with all submissions.

Clinical trial registration	n/a
Study protocol	n/a
Data collection	n/a
Outcomes	n/a

Dual use research of concern

Policy information about dual use research of concern

Hazards

Could the accidental, deliberate or reckless misuse of agents or technologies generated in the work, or the application of information presented in the manuscript, pose a threat to:

No	Yes	
☒	☐	Public health
☒	☐	National security
☒	☐	Crops and/or livestock
☒	☐	Ecosystems
☒	☐	Any other significant area

Experiments of concern

Does the work involve any of these experiments of concern:

No	Yes	
☒	☐	Demonstrate how to render a vaccine ineffective
☒	☐	Confer resistance to therapeutically useful antibiotics or antiviral agents
☒	☐	Enhance the virulence of a pathogen or render a nonpathogen virulent
☒	☐	Increase transmissibility of a pathogen
☒	☐	Alter the host range of a pathogen
☒	☐	Enable evasion of diagnostic/detection modalities
☒	☐	Enable the weaponization of a biological agent or toxin
☒	☐	Any other potentially harmful combination of experiments and agents

Plants

Seed stocks	n/a
Novel plant genotypes	n/a
Authentication	n/a

ChIP-seq

Data deposition

☐ Confirm that both raw and final processed data have been deposited in a public database such as GEO.

☐ Confirm that you have deposited or provided access to graph files (e.g. BED files) for the called peaks.

Data access links<br>*May remain private before publication.*	n/a
Files in database submission	n/a
Genome browser session<br>(e.g. UCSC)	n/a

Methodology

Replicates	n/a
Sequencing depth	n/a
Antibodies	n/a
Peak calling parameters	n/a
Data quality	n/a
Software	n/a

Flow Cytometry

Plots

Confirm that:

- [x] The axis labels state the marker and fluorochrome used (e.g. CD4-FITC).
- [x] The axis scales are clearly visible. Include numbers along axes only for bottom left plot of group (a 'group' is an analysis of identical markers).
- [ ] All plots are contour plots with outliers or pseudocolor plots.
- [x] A numerical value for number of cells or percentage (with statistics) is provided.

Methodology

Sample preparation	For single nucleus genome sequencing, cells were trypsinized, washed in PBS, and resuspended at 20x cell pellet volume in cold nuclear isolation buffer A with digitonin (50 nM HEPES pH 7.3, 150 mM NaCl, 1x dual protease and phosphatase inhibitor, 25g/mL digitonin) by pipetting. The suspensions were rotated at 4°C for 30 minutes, then centrifuged at 500g for 5 minutes at 4°C. Nuclear pellets were washed twice with cold NIB-250 buffer (250 mM sucrose, 15mM Tris-Cl pH 7.5, 60 mM KCl, 15 mM NaCl, 5 mM MgCl2, 1 mM CaCl2) and resuspended in sorting buffer (DPBS with 2% FBS, 0.5 mM spermidine, 500 ng/mL DAPI).<br><br>For the NHEJ reporter assay, cells were collected on day seven after irradiation and analyzed by flow cytometry using a BD LSRFortessa Cell Analyzer.<br><br>For mouse immune profiling, tissues were dissected and placed on ice-cold RPMI supplemented with 10% FBS. Single-cell suspensions of immune cells from the liver were obtained by mechanical disaggregation through a 70µm cell strainer (VWR) and washed through with 10% FBS in RPMI. Liver samples were spun at 60 r.c.f. and 4ºC for 2 min with no brake to pellet hepatocytes before percoll (Cytiva) centrifugation. The supernatant was collected, spun at 420 r.c.f. and 4ºC for 4 min. The pellet was resuspended with 40% Percoll (Cytiva) in HBSS to further remove debris and hepatocytes. The isolated immune cells from the liver went through red blood cell lysis with ACK buffer (KD Medical) before counting cells on a hematocytometer. Splenocytes were isolated by passing cells through a 70µm cell strained followed by red blood cell lysis with ACK buffer before being transferred to a 96-well U-bottom plate and resuspended in fluorescence-activated cell sorting (FACS) buffer (2% FBS in 1X PBS). Viability staining was performed using LIVE/DEAD fixable red stain (1 in 1000 in FACS buffer, Invitrogen) for 15 min at room temperature. Suspensions were then pelleted and resuspended in anti-CD16/32 antibodies (1:500, BioLegend) to block non-specific binding of Fc receptors. Cells were incubated with the indicated surface antibodies for 30 min at 4ºC. a FoxP3 transcription factor staining kit (eBioscience) was used for intracellular staining. Antibodies against intracellular proteins were diluted in 1X permeabilization buffer and added for 45 min at 4ºC. For cytokine staining, cells were stimulated with PMA (final concentration of 1 µg/mL) and ionomycin (Iono, Cell Signaling; final concentration of 1 µg/mL) for 4h at 37ºC in the presence of brefeldin A (GolgiPlug, BD Biosciences; final concentration of 1 µg/mL) to block cytokine export from the golgi apparatus. 2% paraformaldehyde (PFA) was used to fix the cells after staining. Cells were resuspended in 100µL 1X PBS and run on the FACSymphony A3 5-laser flow cytometer (BD Biosciences). Data were analyzed using FlowJo (v.10, BD Biosciences).
Instrument	See above.
Software	FlowJo
Cell population abundance	See above and in manuscirpt.
Gating strategy	See Figs.S3, S7 and S8 for details.

- [x] Tick this box to confirm that a figure exemplifying the gating strategy is provided in the Supplementary Information.

Magnetic resonance imaging

Experimental design

Design type	n/a
Design specifications	n/a
Behavioral performance measures	n/a

Acquisition

Imaging type(s)	n/a
Field strength	n/a
Sequence & imaging parameters	n/a
Area of acquisition	n/a

Diffusion MRI ☐ Used ☐ Not used

Preprocessing

Preprocessing software	n/a
Normalization	n/a
Normalization template	n/a
Noise and artifact removal	n/a
Volume censoring	n/a

Statistical modeling & inference

Model type and settings	n/a
Effect(s) tested	n/a

Specify type of analysis: ☐ Whole brain ☐ ROI-based ☐ Both

Statistic type for inference (See Eklund et al. 2016)	n/a
Correction	n/a

Models & analysis

n/a	Involved in the study
☒	☐ Functional and/or effective connectivity
☒	☐ Graph analysis
☒	☐ Multivariate modeling or predictive analysis
